# Impact of the treatment crossover design on comparative efficacy in EMPOWER-Lung 1: Cemiplimab monotherapy as first-line treatment of advanced non-small cell lung cancer

**DOI:** 10.3389/fonc.2022.1081729

**Published:** 2023-04-04

**Authors:** Josephine Louella Feliciano, Dylan McLoone, Yingxin Xu, Ruben G.W. Quek, Andreas Kuznik, Jean-Francois Pouliot, Giuseppe Gullo, Petra Rietschel, Patricia Guyot, Gerasimos Konidaris, Keith Chan, Sam Keeping, Florence R. Wilson, Nick Freemantle

**Affiliations:** ^1^ Johns Hopkins University, Baltimore, MD, United States; ^2^ PRECISIONheor, Boston, MA, United States; ^3^ Regeneron Pharmaceuticals, Inc., Tarrytown, NY, United States; ^4^ Sanofi, Chilly-Mazarin, France; ^5^ Sanofi, Reading, United Kingdom; ^6^ PRECISIONheor, Vancouver, BC, Canada; ^7^ Institute of Clinical Trials and Methodology, University College London, London, United Kingdom

**Keywords:** non-small cell lung cancer, first-line treatment, cemiplimab, crossover design, EMPOWER-lung 1, chemotherapy

## Abstract

**Objectives:**

In randomized-controlled crossover design trials, overall survival (OS) treatment effect estimates are often confounded by the control group benefiting from treatment received post-progression. We estimated the adjusted OS treatment effect in EMPOWER-Lung 1 (NCT03088540) by accounting for the potential impact of crossover to cemiplimab among controls and continued cemiplimab treatment post-progression.

**Methods:**

Patients were randomly assigned 1:1 to cemiplimab 350 mg every 3 weeks (Q3W) or platinum-doublet chemotherapy. Patients with disease progression while on or after chemotherapy could receive cemiplimab 350 mg Q3W for ≤108 weeks. Those who experienced progression on cemiplimab could continue cemiplimab at 350 mg Q3W for ≤108 additional weeks with four chemotherapy cycles added. Three adjustment methods accounted for crossover and/or continued treatment: simplified two-stage correction (with or without recensoring), inverse probability of censoring weighting (IPCW), and rank-preserving structural failure time model (RPSFT; with or without recensoring).

**Results:**

In the programmed cell death-ligand 1 ≥50% population (N=563; median 10.8-month follow-up), 38.2% (n=107/280) crossed over from chemotherapy to cemiplimab (71.3%, n=107/150, among those with confirmed progression) and 16.3% (n=46/283) received cemiplimab treatment after progression with the addition of histology-specific chemotherapy (38.7%, n=46/119, among those with confirmed progression). The unadjusted OS hazard ratio (HR) with cemiplimab versus chemotherapy was 0.566 (95% confidence interval [CI]: 0.418, 0.767). Simplified two-stage correction—the most suitable method based on published guidelines and trial characteristics—produced an OS HR of 0.490 (95% CI: 0.365, 0.654) without recensoring and 0.493 (95% CI: 0.361, 0.674) with recensoring. The IPCW and RPSFT methods produced estimates generally consistent with simplified two-stage correction.

**Conclusions:**

After adjusting for treatment crossover and continued cemiplimab treatment after progression with the addition of histology-specific chemotherapy observed in EMPOWER-Lung 1, cemiplimab continued to demonstrate a clinically important and statistically significant OS benefit versus chemotherapy, consistent with the primary analysis.

## Introduction

Cemiplimab (cemiplimab-rwlc in the United States) is a highly potent, hinge-stabilized, immunoglobulin G4 fully human monoclonal antibody directed against the programmed cell death-1 (PD-1) receptor. Cemiplimab was approved in 2021 in the United States as a first-line monotherapy for treatment of patients with metastatic or locally advanced non-small cell lung cancer (NSCLC) who are not candidates for surgical resection or definitive chemoradiation, and whose tumors have high programmed cell death-ligand 1 (PD-L1) expression (tumor proportion score ≥50%) with no epidermal growth factor receptor (*EGFR*), anaplastic lymphoma kinase (*ALK*), or c-ros oncogene 1 (*ROS1*) genomic aberrations (1–3). This approval was based on published data from EMPOWER-Lung 1: a phase 3, multicenter, open-label, global randomized-controlled trial comparing cemiplimab monotherapy with investigator’s choice of platinum-doublet chemotherapy for the first-line treatment of patients with advanced NSCLC whose tumors express PD-L1 ≥50% (1). Treatment with cemiplimab resulted in a statistically significant improvement in overall survival (OS) and progression-free survival, reducing the risk of death by 43.4% versus investigator’s choice of platinum-doublet chemotherapy. The safety profile was consistent with previously reported data for cemiplimab and other PD-1 or PD-L1 inhibitors (1). This OS improvement was observed despite the treatment crossover design, in which patients in the chemotherapy arm were allowed to cross over from chemotherapy to cemiplimab upon disease progression. Patients in the cemiplimab arm were also allowed to continue cemiplimab with the addition of four cycles of histology-specific chemotherapy upon disease progression (this was a protocol amendment introduced after the start of the study).

In a recent network meta-analysis of immunotherapies in first-line advanced NSCLC with PD-L1 ≥50% (4), the base case results comparing EMPOWER-Lung 1 (1), KEYNOTE-024 (5), and KEYNOTE-042 (6) demonstrated cemiplimab was associated with comparable OS and statistically significant improvements in progression-free survival from 6–30 months versus pembrolizumab. No statistically significant differences in the incidence of grade 3–5 adverse events (AEs), grade 3–5 immune-mediated AEs, and all-cause discontinuations due to AEs were observed between the two treatments. However, a limitation of this analysis was that crossover in EMPOWER-Lung 1 was not accounted for in the network meta-analysis, though it is a potential confounding variable that could impact OS.

In randomized-controlled trials with treatment crossover designs (i.e., patients are permitted to cross over from the control treatment to the experimental treatment or switch to subsequent treatment when an event such as disease progression occurs), the benefit received from the new treatment post-progression often results in an underestimation of the relative treatment effect of OS (7, 8). In these situations, adjusting for the potential bias in OS introduced by treatment crossover is critical when making clinical decisions and optimizing treatment sequencing for patients (7, 8).

The objective of this study was to estimate the adjusted OS treatment effect in EMPOWER-Lung 1 by accounting for confounding associated with patients who crossed over from chemotherapy to cemiplimab per the study design, and who received cemiplimab continuation with the addition of chemotherapy per protocol amendment.

## Materials and methods

### EMPOWER-Lung 1 study design

As previously reported (9), EMPOWER-Lung 1 (NCT03088540) included adults (age ≥18 years) with histologically or cytologically confirmed stage IIIB or IIIC (not candidates for definitive chemoradiation therapy) or previously untreated stage IV squamous or non-squamous NSCLC with PD-L1 expressed on ≥50% of tumor cells, measured using the PD-L1 immunohistochemistry 22C3 pharmDx assay. Prior adjuvant or neoadjuvant chemotherapy was permitted, provided that patients developed recurrent or metastatic disease >6 months after completing therapy. Prior cytotoxic T-lymphocyte-associated antigen-4 (CTLA-4) treatment was allowed, provided that the last dose of such an antibody was ≥3 months before the first dose of study drug. Additional eligibility criteria included an Eastern Cooperative Oncology Group (ECOG) performance status of 0 or 1; adequate organ and bone marrow function; and presence of at least one measurable lesion per the Response Evaluation Criteria in Solid Tumors version 1.1 (RECIST 1.1). Patients were ineligible if they had never smoked (defined as ≤100 cigarettes in a lifetime) or had active or untreated brain metastases.

Patients were randomly assigned 1:1 to receive either cemiplimab 350 mg administered intravenously over a period of 30 minutes every 3 weeks for ≤108 weeks (i.e., ≤36 treatment cycles) or four to six cycles of investigator’s choice of platinum-doublet chemotherapy. Cemiplimab dose modification was not allowed but chemotherapy dose modification was allowed. Maintenance pemetrexed was permitted for patients with non-squamous histology. Treatments were continued for the specified duration until disease progression as defined per RECIST 1.1.

**Figure 1:**
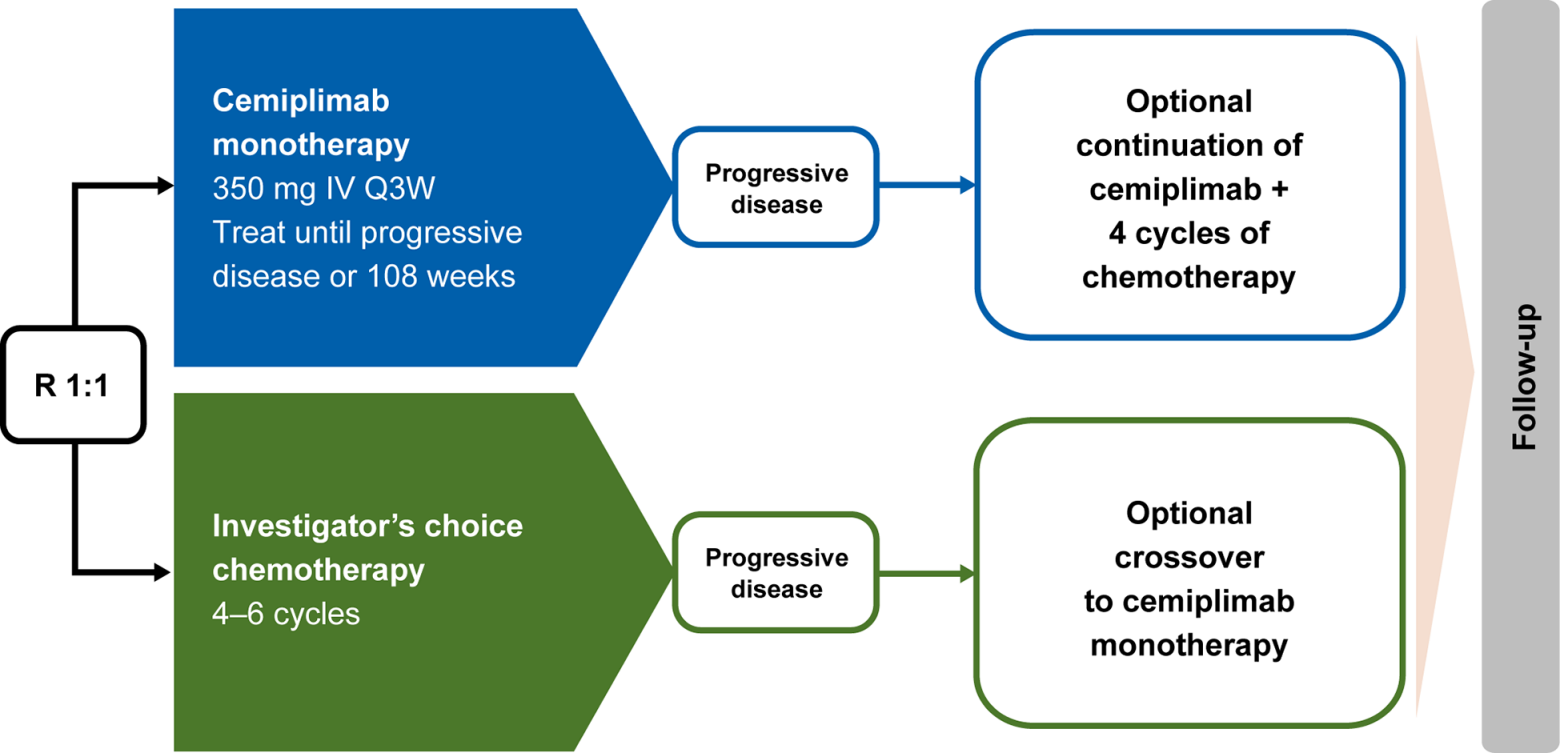
EMPOWER-Lung 1 treatment crossover trial design. Conditions for crossover or continued treatment in both trial arms included independent review committee–assessed RECIST 1.1–defined disease progression. IV, intravenous; Q3W, every 3 weeks; R, randomized; RECIST 1.1, Response Evaluation Criteria in Solid Tumors, version 1.1.

In EMPOWER-Lung 1, patients who experienced disease progression while on or after completion of chemotherapy were offered the option to receive cemiplimab 350 mg every 3 weeks for ≤108 weeks, provided they met all the inclusion criteria for cemiplimab therapy: independent review committee–confirmed disease progression per RECIST 1.1; use of a PD-1 inhibitor assessed by investigator as an appropriate second-line treatment; and patient continued to meet all other study eligibility criteria, as defined in the inclusion and exclusion criteria. Intolerance to chemotherapy was not allowed as a reason to cross over. Based on a protocol amendment, patients who experienced disease progression while receiving cemiplimab based on RECIST 1.1 criteria could continue treatment with cemiplimab 350 mg every 3 weeks for ≤108 additional weeks, along with the addition of histology-specific chemotherapy for four cycles, provided they met the aforementioned criteria, had not completed the 108-week treatment period, were tolerant of cemiplimab, and had a stable ECOG performance status. Alternatively, these patients could opt to initiate a new anticancer treatment, which was most often a chemotherapy-based regimen. This aligns with the current National Comprehensive Cancer Network guidelines which state that patients who experience disease progression on PD-1/PD-L1 inhibitors are not recommended to switch to another PD-1/PD-L1 inhibitor (10). A simplified overview of the options for crossover and continued treatment of cemiplimab in addition to chemotherapy is presented in **Figure 1**.

### Statistical analysis

Three adjustment methods were applied to account for treatment crossover and/or continued treatment in the EMPOWER-Lung 1 trial: (i) simplified two-stage correction with and without recensoring, (ii) inverse probability of censoring weighting (IPCW), and (iii) a rank-preserving structural failure time (RPSFT) model with and without recensoring.

The simplified two-stage correction was designed according to the treatment crossover commonly observed in oncology trials (i.e., when switching occurs after a disease-related time point) and can account for both treatment crossover and continued treatment (8, 11). In stage 1, various parametric survival models were fitted to estimate the effect of crossover in the chemotherapy arm and the effect of continued treatment in the cemiplimab arm, with the log-normal distribution selected as the best fitting. The models were then adjusted for the following covariates which were validated by clinicians: sex, age at baseline, brain metastases at baseline and disease progression, liver metastases at baseline and disease progression, and ECOG performance status at baseline and disease progression. Patients were considered eligible for inclusion in stage 1 if they met the eligibility criteria for treatment crossover (i.e., had independent review committee–confirmed progressive disease, had a stable ECOG performance status of 0 or 1 at disease progression, and continued to meet other study eligibility criteria). The effects of crossover and continued treatment estimated from the accelerated failure time models (i.e., acceleration factors) were used to adjust survival times among patients who switched treatments. In stage 2, a Cox proportional hazards model was fitted to compare the adjusted survival times that were estimated in stage 1. Bootstrapping was used to estimate the 95% confidence interval (CI) of the acceleration factor and treatment effect hazard ratio (HR) of cemiplimab versus investigator’s choice of platinum-doublet chemotherapy. Analyses were conducted both with and without recensoring applied (i.e., data censored at earlier timepoints to avoid informative censoring). Although recensoring can avoid the bias introduced by the association between adjusted censoring times and prognosis, it often results in a loss of follow-up (8).

In the IPCW approach (12), patients were artificially censored at the time of crossover or continued treatment. To mitigate selection bias, the remaining observations were weighted based on time-varying demographic and disease characteristics. Stabilized weights were calculated using a mixed-effects logistic regression, adjusted for the following covariates: sex, age at baseline, brain metastases over time, liver metastases over time, and ECOG performance status over time. In cases of missing covariate values over time, such as ECOG performance status, the last recorded measurement before a given timepoint was used. The presence or absence of brain and liver metastases were evaluated at baseline and assumed to remain the same unless otherwise reported in the data. A weighted Cox proportional hazards model was then fitted to estimate an HR, with the 95% CI estimated by bootstrapping.

In the RPSFT model (13), a multiplicative acceleration factor, stratified by histology, was determined using G-estimation which was used to adjust the survival times of patients who had crossed over during the study. Bootstrapping was used to estimate the 95% CI of the acceleration factor. Analyses were conducted both with and without recensoring applied to the survival times of patients in the chemotherapy arm. A Cox proportional hazards model stratified by histology was fitted to estimate an HR and 95% CI of observed survival in the cemiplimab arm versus adjusted survival in the chemotherapy arm.

The simplified two-stage correction and IPCW methods could be applied to both treatment arms (i.e., crossover and continued treatment), while the RPSFT model could only adjust for crossover, not continued treatment. Using published guidance on appropriate methods for accounting for treatment switching (14), the simplified two-stage correction was considered the most suitable for this analysis as it was designed to account for treatment crossover often observed in oncology trials (8, 11). In this method, at the point of disease progression, the effect of the new treatment on extending survival from the point of disease progression to death can be estimated for patients who switched treatments. The RPSFT and IPCW methods were used to support and validate findings from the simplified two-stage correction.

## Results

### Patients and treatment

In the PD-L1 ≥50% population (N=563) of EMPOWER-Lung 1, at a median 10.8 months of follow-up (data cutoff: March 1, 2020), the proportion of patients in the chemotherapy arm who crossed over to receive cemiplimab was 38.2% (n=107/280, with a median of five crossover treatment cycles; range: 1–29; mean: 6.36), which corresponded to 71.3% (n=107/150) of patients who progressed on chemotherapy. Another five patients who progressed on chemotherapy but did not cross over subsequently received varied chemotherapy regimens (n=3) or pembrolizumab regimens (n=2). The proportion of patients in the cemiplimab arm who received continued treatment with cemiplimab in addition to chemotherapy was 16.3% (n=46/283, with a median of four continued treatment cycles; range: 1–13; mean: 4.98), which corresponded to 38.7% (n=46/119) of patients who progressed on cemiplimab. An additional 14 patients who progressed on cemiplimab but did not receive continued treatment with cemiplimab in addition to chemotherapy were subsequently treated with chemotherapy regimens (n=11), bevacizumab regimens (n=1), or other targeted therapies (n=2).

Of those who crossed over from chemotherapy to cemiplimab (n=107), median age (range) was 62 (40–81) years, 11.2% were female, and 75.7% had an ECOG performance status of 1. Similar characteristics were observed in patients who received continued treatment with cemiplimab in addition to chemotherapy (n=46; **Table 1**).

**Table 1:** Demographic and baseline disease characteristics among the EMPOWER-Lung 1 PD-L1 ≥50% population.

Characteristic	Cemiplimab			Chemotherapy		
	Randomized to cemiplimab (n=283)	Continued treatment from cemiplimab to cemiplimab + chemotherapy (n=46)	Did not receive continued treatment (n=237)	Randomized to chemotherapy (n=280)	Crossed over from chemotherapy to cemiplimab (n=107)	Without crossover (n=173)
Age, median (range), years	63 (31–79)	62.5 (43–75)	63 (31–79)	64 (40–84)	62 (40–81)	65 (46–84)
Female, n (%)	35 (12.4)	5 (10.9)	30 (12.7)	49 (17.5)	12 (11.2)	37 (21.4)
ECOG performance status, n (%)						
0	77 (27.2)	14 (30.4)	63 (26.6)	75 (26.8)	26 (24.3)	49 (28.3)
1	206 (72.8)	32 (69.6)	174 (73.4)	205 (73.2)	81 (75.7)	124 (71.7)
Region of enrollment, n (%)						
East Asia	31 (11.0)	8 (17.4)	23 (9.7)	29 (10.4)	13 (12.1)	16 (9.2)
Not East Asia	252 (89.0)	38 (82.6)	214 (90.3)	251 (89.6)	94 (87.9)	157 (90.8)
Histology, n (%)						
Squamous	122 (43.1)	26 (56.5)	96 (40.5)	121 (43.2)	55 (51.4)	66 (38.2)
Non-squamous	161 (56.9)	20 (43.5)	141 (59.5)	159 (56.8)	52 (48.6)	107 (61.8)
Smoking status, n (%)						
Current	105 (37.1)	22 (47.8)	83 (35.0)	92 (32.9)	46 (43.0)	46 (26.6)
Past	178 (62.9)	24 (52.2)	154 (65.0)	188 (67.1)	61 (57.0)	127 (73.4)
Brain metastases, n (%)	34 (12.0)	3 (6.5)	31 (13.1)	34 (12.1)	13 (12.1)	21 (12.1)
Liver metastases, n (%)	49 (17.3)	8 (17.4)	41 (17.3)	44 (15.7)	14 (13.1)	30 (17.3)

ECOG, Eastern Cooperative Oncology Group; PD-L1, programmed cell death-ligand 1.

### Outcomes

**Figure 2:**
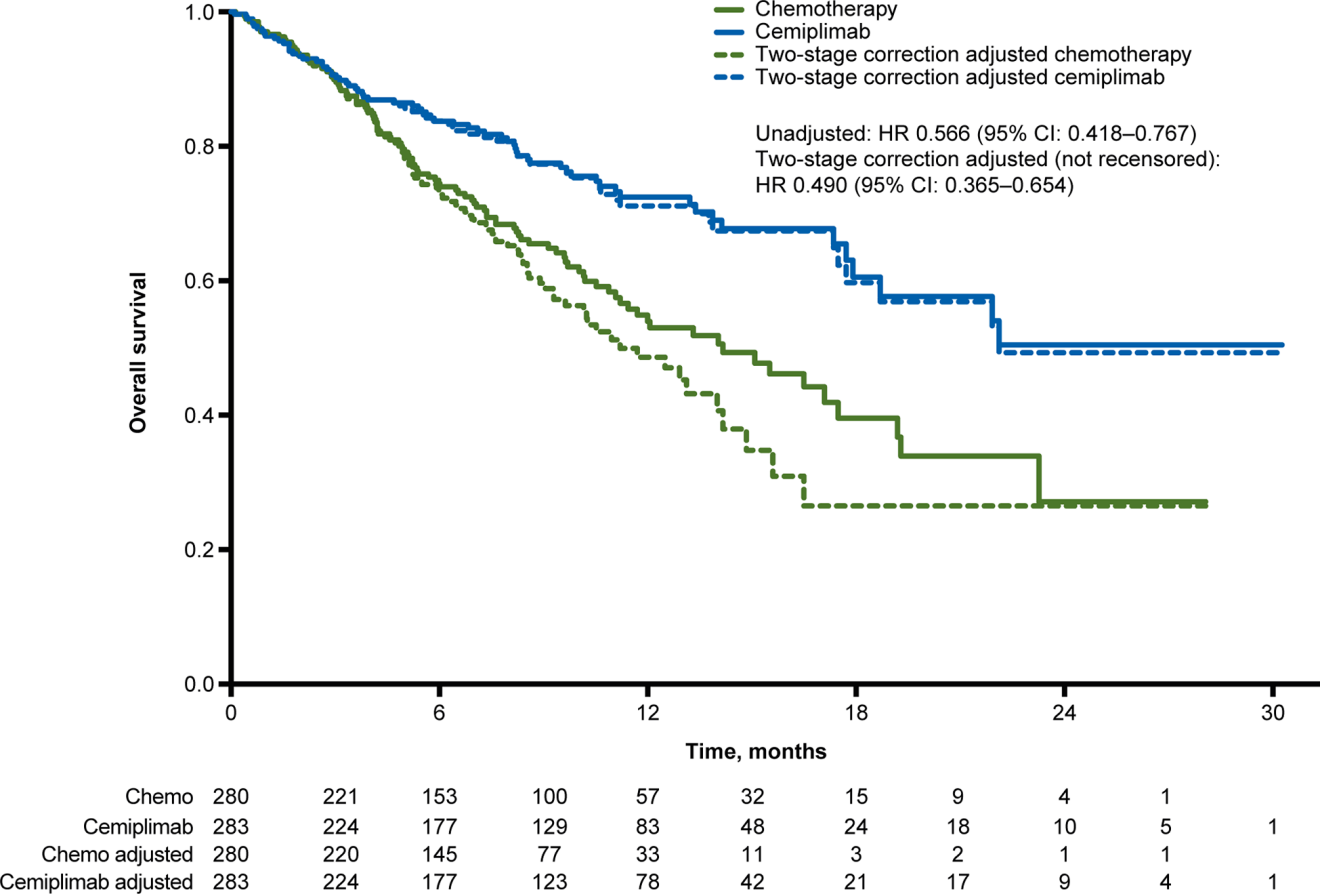
Overall survival Kaplan-Meier curves for the unadjusted and crossover-adjusted results from the EMPOWER-Lung 1 PD-L1 ≥50% population based on simplified two-stage correction. Values under the figure represent number of patients at risk. Chemo, chemotherapy; CI, confidence interval; HR, hazard ratio; PD-L1, programmed cell death-ligand 1.

At median follow-up of 10.8 months, the unadjusted OS HR of cemiplimab versus chemotherapy was 0.566 (95% CI: 0.418, 0.767). The simplified two-stage correction produced an OS HR of 0.490 (95% CI: 0.365, 0.654) without recensoring and 0.493 (95% CI: 0.361, 0.674) when recensoring was incorporated (**Figure 2**). The IPCW estimate of the OS HR was 0.571 (95% CI: 0.413, 0.802). With the RPSFT without recensoring the OS HR was 0.470 (95% CI: 0.343, 0.643) and with recensoring the OS HR was 0.499 (95% CI: 0.348, 0.715; **Table 2**).

**Table 2:** Crossover adjustment results among the EMPOWER-Lung 1 PD-L1 ≥50% population.

Crossover adjustment method	Median OS, months (95% CI)		OS HR (95% CI) for cemiplimab versus chemotherapy
	Cemiplimab (n=283)	Chemotherapy (n=280)	
None	NR (17.9, NE)	14.2 (11.2, 17.5)	0.566 (0.418, 0.767)
Simplified two-stage correction (no recensoring)	22.1 (17.7, NE)	11.2 (9.3, 14.2)	0.490 (0.365, 0.654)
Simplified two-stage correction (with recensoring)	NR (17.7, NE)	11.2 (9.3, 14.2)	0.493 (0.361, 0.674)
IPCW	21.9 (17.7, NE)	11.7 (9.6, 16.5)	0.571 (0.413, 0.802)
RPSFT (no recensoring)	NR (17.9, NE)	11.3 (9.6, 15.0)	0.470 (0.343, 0.643)
RPSFT (with recensoring)	NR (17.9, NE)	11.0 (8.7, NE)	0.499 (0.348, 0.715)

Based on median follow-up of 10.8 months. CI, confidence interval; HR, hazard ratio; IPCW, inverse probability of censoring weighting; NE, not evaluable; NR, not reached; OS, overall survival; RPSFT, rank-preserving structural failure time.

## Discussion

Treatment crossover is common in oncology trials, and its implications should be considered when interpreting evidence of the clinical benefit associated with new treatments. Specifically, treatment crossover poses a risk of bias as the introduction of the new treatment post-progression can lessen the observed relative treatment effects between interventions versus what would have been observed had no crossover taken place. After adjusting for the treatment crossover and continued treatment in EMPOWER-Lung 1, cemiplimab demonstrated a clinically meaningful and statistically significant OS benefit that was consistent with the results of the primary unadjusted analysis.

Three common statistical methods were used to adjust for the influence of treatment crossover on OS, and all three estimated HRs strongly favored cemiplimab treatment. The simplified two-stage correction was preferred, since the RPSFT method did not allow for adjustment of continued treatment based on standard applications, and\ the IPCW method relies on stronger assumptions and is more prone to bias in relatively small sample sizes (11). Both the IPCW and simplified two-stage correction methods rely on the assumption of no unmeasured confounders. For this analysis, in cases of missing covariate values over time, the last recorded measurement before a given timepoint was used. Since the IPCW method requires that the covariates be measured consistently over time, there were more instances of using the last recorded covariate measurement which could have introduced more uncertainty from the covariates than in the simplified two-stage correction.

All three methods produced consistent treatment effect estimates, demonstrating the results were not sensitive to the adjustment method applied to the EMPOWER-Lung 1 data set. The IPCW method resulted in a slightly more conservative HR, which may have been due to its ability to account for time-dependent confounding; however, as outlined above, the simplified two-stage correction was still preferred as this approach relied on fewer assumptions. In all three methods, after accounting for crossover from chemotherapy to cemiplimab and continued treatment of cemiplimab in addition to chemotherapy, the substantial and statistically significant OS benefit of cemiplimab versus chemotherapy was confirmed.

The methods applied and corresponding results were similar to those observed for other trials in this population which allowed crossover. In the KEYNOTE-024 trial evaluating pembrolizumab, patients in the chemotherapy arm who experienced disease progression were permitted to cross over to pembrolizumab (15). Similar to the current study, Reck et al. used three statistical methods to adjust for the influence of crossover on OS, and all three methods supported an HR more strongly favoring pembrolizumab (5). The simplified two-stage correction was preferred, given the lack of supporting evidence for a common treatment effect as assumed in RPSFT models and because the IPCW method can be more prone to bias in relatively small sample sizes (5).

A strength of the current analysis is the consistency of results by all three methods indicating the robustness of crossover adjustment analyses. The EMPOWER-Lung 1 crossover adjustment accounts not only for the potential bias introduced by crossover from chemotherapy to cemiplimab but also for the potential bias of continued treatment with cemiplimab in addition to chemotherapy. This was an exploratory analysis with the primary limitation being the length of follow-up (median follow-up of 10.8 months) in EMPOWER-Lung 1 at the time of analysis. Crossover adjusted analyses for EMPOWER-Lung 1 may be updated once more mature data become available.

After accounting for treatment crossover and continued treatment observed in EMPOWER-Lung 1, cemiplimab continued to demonstrate a clinically important and statistically significant OS benefit versus chemotherapy, consistent with the primary analysis.

## Data availability statement

Qualified researchers may request access to study documents (including the clinical study report, study protocol with any amendments, blank case report form, statistical analysis plan) that support the methods and findings reported in this manuscript. Individual anonymized participant data will be considered for sharing once the product and indication has been approved by major health authorities (e.g., FDA, EMA, PMDA, etc.), if there is legal authority to share the data and there is not a reasonable likelihood of participant re-identification. Submit requests to https://vivli.org/.

## Ethics statement

The EMPOWER-Lung 1 study protocol and all amendments have been approved by the institutional review boards or independent ethics committees at each participating study site. The study was conducted in accordance with the Declaration of Helsinki and the International Conference on Harmonisation Good Clinical Practice guidelines. Full details of the ethical approval can be found in the **Supplementary Material**. All patients provided written informed consent before enrolment.

## Author contributions

All authors contributed to the conception and design of the work and were involved in the analysis and interpretation of data. DM and KC performed the statistical analysis. All authors were responsible for providing critical guidance, and drafting/revising the manuscript. All authors contributed to the article and approved the submitted version.

## References

[1] Sezer A Kilickap S Gümüş M Bondarenko I Özgüroğlu M Gogishvili M. Cemiplimab monotherapy for first-line treatment of advanced non-small-cell lung cancer with PD-L1 of at least 50%: A multicentre, open-label, global, phase 3, randomised, controlled trial. Lancet (2021) 397(10274):592–604. doi: 10.1016/s0140-6736(21)00228-2 33581821

[2] Regeneron Pharmaceuticals, Inc. FDA approves Libtayo® (cemiplimab-rwlc) monotherapy for patients with first-line advanced non-small cell lung cancer with PD-L1 expression of ≥50% (2021). Available at: https://investor.regeneron.com/news-releases/news-release-details/fda-approves-libtayor-cemiplimab-rwlc-monotherapy-patients-first.

[3] Regeneron Pharmaceuticals, Inc. LIBTAYO® (cemiplimab-rwlc) injection, for intravenous use [US prescribing information] (2021). Available at: https://www.accessdata.fda.gov/drugsatfda_docs/label/2021/761097s007lbl.pdf.

[4] Freemantle N Xu Y Wilson FR Guyot P Chen CI Keeping S. Network meta-analysis of immune-oncology monotherapy as first-line treatment for advanced non-small-cell lung cancer in patients with PD-L1 expression ≥50. Ther Adv Med Oncol (2022) 14:17588359221105024. doi: 10.1177/17588359221105024 35747163 PMC9210099

[5] Reck M Rodriguez-Abreu D Robinson AG Hui R Csoszi T Fulop A. Updated analysis of KEYNOTE-024: Pembrolizumab versus platinum-based chemotherapy for advanced non-small-cell lung cancer with PD-L1 tumor proportion score of 50% or greater. J Clin Oncol (2019) 37(7):537–46. doi: 10.1200/JCO.18.00149 30620668

[6] Mok TSK Wu YL Kowalski DM Turna HZ Laktionov KK Lubiniecki GM. Pembrolizumab versus chemotherapy for previously untreated, PD-L1-expressing, locally advanced or metastatic non-small-cell lung cancer (KEYNOTE-042): A randomised, open-label, controlled, phase 3 trial. Lancet (2019) 393(10183):1819–30. doi: 10.1016/S0140-6736(18)32409-7 30955977

[7] Latimer NR Abrams KR. NICE DSU technical support document 16: Adjusting survival time estimates in the presence of treatment switching: report by the decision support unit. United Kingdom: School of Health and Related Research, University of Sheffield, UK; Department of Health Sciences, University of Leicester, UK (2014). 27466662

[8] Latimer NR Abrams KR Lambert PC Crowther MJ Wailoo AJ Morden JP. Adjusting survival time estimates to account for treatment switching in randomized controlled trials–an economic evaluation context: Methods, limitations, and recommendations. Med Decis Making (2014) 34(3):387–402. doi: 10.1177/0272989x13520192 24449433

[9] Sezer A Kilickap S Gümüş M Bondarenko I Özgüroğlu M Gogishvili M. Cemiplimab monotherapy for first-line treatment of advanced NSCLC with PD-L1 ≥50%: a randomised controlled trial. Lancet (2021) 397(10274):592–604. doi: 10.1016/S0140-6736(21)00228-2 33581821

[10] National Comprehensive Cancer Network (NCCN). NCCN clinical practice guidelines in oncology: Non-small cell lung cancer. (2022). Available at: https://www.nccn.org/professionals/physician_gls/PDF/nscl.pdf.

[11] Latimer NR Abrams KR Lambert PC Crowther MJ Wailoo AJ Morden JP. Adjusting for treatment switching in randomised controlled trials - a simulation study and a simplified two-stage method. Stat Methods Med Res (2017) 26(2):724–51. doi: 10.1177/0962280214557578 25416688

[12] Robins JM Finkelstein DM. Correcting for noncompliance and dependent censoring in an AIDS clinical trial with inverse probability of censoring weighted (IPCW) log-rank tests. Biometrics (2000) 56:779–88. doi: 10.1111/j.0006-341x.2000.00779.x 10985216

[13] Robins JM Tsiatis AA. Correcting for non-compliance in randomized trials using rank preserving structural failure time models. Commun Stat Theory Methods (1991) 20:2609–31. doi: 10.1080/03610929108830654

[14] Sullivan TR Latimer NR Gray J Sorich MJ Salter AB Karnon J. Adjusting for treatment switching in oncology trials: A systematic review and recommendations for reporting. Value Health (2020) 23(3):388–96. doi: 10.1016/j.jval.2019.10.015 32197735

[15] Reck M Rodriguez-Abreu D Robinson AG Hui R Csoszi T Fulop A. Pembrolizumab versus chemotherapy for PD-L1-positive non-small-cell lung cancer. N Engl J Med (2016) 375(19):1823–33. doi: 10.1056/NEJMoa1606774 27718847

