# Supplementary material for: Impact of the treatment crossover design on comparative efficacy in EMPOWER-Lung 1: Cemiplimab monotherapy as first-line treatment of advanced non-small cell lung cancer
Source: Front Oncol. 2023 Apr 4;12:1081729. doi: 10.3389/fonc.2022.1081729 (PMC10110970; doi:10.3389/fonc.2022.1081729)
Supplement: Supplementary file 1 [file Table_1.pdf]

Supplementary Table. List of institutional review boards and independent ethics committees that approved the clinical trial protocol

Country	Study site no.	Local institutional review boards and independent ethics committees	Central institutional review boards and independent ethics committees
Australia	36001		Bellberry Human Research Ethics Committee 129 Glen Osmond Road Eastwood, South Australia 5063
Australia	36002		Bellberry Human Research Ethics Committee 129 Glen Osmond Road Eastwood, South Australia 5063
Brazil	76001	Comitê de Ética em Pesquisa do Hospital Santa Izabel - Santa Casa de Misericórdia da Bahia / Prof. Dr. Celso Figueirôa Praça Conselheiro Almeida Couto , 500 – Nazaré Salvador, Bahia, Brazil 40050-410	
Brazil	76002	Comitê de Ética em Pesquisa do Hospital de Clínicas de Porto Alegre - HCPA / UFRGS Rua Avelino Tallini, ,171 - Sala 309 - Prédio 01 ,Bairro Rua Ramiro Barcelos, 2350 - 2° andar ,2227 F Porta Alegra, Rio Grande do Sul, Brazil	
Brazil	76003	Comitê de Ética em Pesquisa do Centro Universitário UNIVATES Rua Avelino Tallini, ,171 - Sala 309 - Prédio 01 ,Bairro Universitário Lejaedo Rio Grande do Sul, Brazil 95914-014	
Brazil	76004	Comitê de Ética em Pesquisa Hospital Municipal São José / HMSJ Joinville – SC Avenida Getúlio Vargas, 238 Joinville, Santa Catarina, Brazil 89202-050	
Brazil	76006	Comitê de Ética em Pesquisa do Instituto de Assistência Médica Ao Servidor Público Estadual - IAMSPE – SP Av. Ibirapuera, 981, 1º andar - sala 106, Prédio da Administração Sao Paulo, Sao Paulo Brazil 04029-000	

Brazil	76008	Comitê de Ética em Pesquisa da Universidade de Mogi das Cruzes - UMC/SP Av. Cândido Xavier de Almeida Souza, 200 - Prédio II, sala 2121 - Centro Cívico Mogi Das Cruzes, Sao Paulo, Brazil 08780-911	
Brazil	76013	Comitê de Ética em Pesquisa da Fundação Pio XII - Hospital de Câncer de Barretos Rua Antenor Duarte Vilela, 1331. Dr Paulo Prata Barretos, sao Paulo, Brazil 14784-400	CONEP (Comissão Nacional de Ética em Pesquisa) SRTVN 701, Via W 5 Norte, lote D - Edifício PO 700, 3º andar/ Asa Norte Sofia, Brasilia, Distrito Federal, Brazil 70719-040
Brazil	76015	Comitê de Ética em Pesquisa da Universidade de Passo Fundo/ Pró-Reitoria de Pesquisa e Pós-Graduação - VRPPG/ UPF BR 285, Km 292, Campus I - Centro Administrativo/Reitoria 4 andar - São José, paso Fundo, Rio Grande do Sul, Brazil 99052-900	
Brazil	76016	Comitê de Ética em Pesquisa da Liga Paranaense de Combate ao Câncer Rua Dr. Ovande do Amaral, 201 - ,Jardim das Américas Curitiba Paraná Brazil, 81520-060	
Brazil	76017	Comitê de Ética em Pesquisa da Universidade de Passo Fundo/ Pró-Reitoria de Pesquisa e Pós-Graduação - VRPPG/ UPF Praça Piratinino de Almeida, 53 – Centro Pelotas, Rio Grande do Sul, Brazil 96015- 290	
Brazil	76018	Comitê de Ética em Pesquisa do Hospital Pró-Cardíaco - ESHO Empresa de Serviços Hospitalares / HPC Rua Voluntários da Pátria, 435, 8º andar – Botafogo, Rio de Janeiro, Brazil 22270-005	
Brazil	76019	Comitê de Ética em Pesquisa do Instituto de Medicina Integral Professor Fernando Figueira - IMIP/PE Rua dos Coelhos, 300 - Prédio Administrativo Orlando Onofre, 1º Andar. Boa Vista Recife, Brazil 50070-555	

Brazil	76022	Comitê de Ética em Pesquisa do Instituto Brasileiro de Controle do Câncer – IBCC<br>Avenida Alcântara Machado, 2576 – Mooca São Paulo, São Paulo, Brazil 03102-002	
Bulgaria	100001		Ethics Committee for Multicenter Trials<br>8 Damian Gruev<br>Sofia, Bulgaria 1303
Bulgaria	100002		Ethics Committee for Multicenter Trials<br>8 Damian Gruev<br>Sofia, Bulgaria 1303
Belarus	112001	Ethics Committee at Healthcare Institution 'Mogilev Regional Oncology Dispensary'<br>Pavlova Street, 2a<br>Mogilev,Belarus<br>212018	
Belarus	112002	Ethics Committee at Minsk City Clinical Oncology Dispensary<br>64, Nezavisimosti Ave.<br>Minsk, Belarus<br>220113	
Chile	152001	Villar, Hector Camerati<br>Comité Ético Cientifico Servicio de Salud de Viña, Calle Limache 1307 2º Piso, Vina Del Mar, Chile 2520563	
Chile	152002	Comité de Ética de la Investigacion del Servicio<br>Calle San Jose 1053<br>Santiago, Chile 8380755	
Chile	152004	Comité Ético Científico Clínica Reñaca<br>Anabaena 336, Jardín del Mar, Vina Del Mar<br>Chile 2540364<br>Comité Ético Cientifico Servicio de Salud de Viña, Calle Limache 1307 2º Piso, Vina Del Mar, Chile 2520563	
Chile	152005	Comité de Ética de la Investigacion del Servicio, Calle San Jose 1053, Santiago, Chile 8380755	
China	156001	Ethics Review Committee of Shanghai Pulmonary Hospital<br>No. 507, Zhengmin Road, Yangpu District,<br>Shanghai City, China<br>200433	

China	156003	Ethics Committee of Linyi Cancer Hospital<br>No.6 East Lingyuan Road, Lanshan District,<br>Linyi, China 276000	
China	156004	Medical Ethic Committee of Beijing Cancer Hospital<br>No.81 Fu-cheng Road, Haidian District<br>Beijing, China 100142	
China	156017	Drug Ethics Committee of Tianjin Medical University General Hospital<br>No.154 Anshan Avenue, Tianjin, China<br>300052	
Taiwan	158001	Taipei Medical University-Joint Institutional Review Board 17F., No. 172-1, Sec. 2, Keelung Rd., Daan District, Taipei, Taiwan 110	
Taiwan	158002	Institutional Review Board of Taichung Veterans General Hospital<br>1650 Taiwan Boulevard Sect. 4, Taichung, Taiwan 40705	
Taiwan	158003	Institutional Review Board, Taipei Vetrans General<br>201, Shih-Pai Road, Sec. 2, Taipei, Taiwan<br>11217	
Taiwan	158004	Institutional Review Board, Kaohsiung Medical University Chung-Ho Memorial Hospital NO. 100, Tzyou 1st Road, Kaohsiung, Taiwan 807	
Taiwan	158005	Research Ethics Committtee, China Medical University Hospital, 2 Yude Road, Taichung, Taiwan<br>40447	
Taiwan	158013	Institutional Review Board, E-DA Hospital<br>No. 6, Yida Rd., Yanchao Dist, Kaohsiung City, Taiwan 82445	
Colombia	170001	Comité de Ética en Investigaciòn Clínica de la Costa LTDA:<br>Carrera 50 # 80-144.,<br>Barranquilla, Atlantico, Colombia, 080020	
Colombia	170004	Comité de Ética en Investigación CEI FOSCAL	

		Calle 158 # 20-95.<br>FOSUNAB, Piso 2.<br>Flordiablanca, Santander,<br>Colombia<br>681004	
Czech Republic	203001	Eticka komise IKEM a TN<br>Videnska 800<br>Praha, Praha 4<br>Czech Republic 14059	
Czech Republic	203004	Eticka komise VFN v Praze<br>Na Bojisti 1, III. Patro<br>Praha 2<br>Czech Republic 12808	
Georgia	268002	Independent Ethics<br>Committee of LTD "High<br>Technology Medical Center,<br>University Clinic"<br>9, tsinandali Str., Tbilisi,<br>Georgia 0144	
Georgia	268004	Independent Ethics<br>Committee of LTD “High<br>Technology Hospital<br>Medcenter”<br>118, Pushkin Str., Batumi,<br>Georgia 6000	
Georgia	268005	Independent Ethics<br>Committee of Acad. Fridon<br>Todua Medical Center –<br>Research Institute of Clinical<br>Medicine<br>13, Tevdrore Mgvdelis str.,<br>Tbilisi, Georgia 0112	
Georgia	268006	Independent Ethics<br>Committee of JSC 'Neo Medi'<br>12, Kristine Sharashidze Str.,<br>Tbilisi, Georgia 0159	
Georgia	268007	Independent Ethics<br>Committee of LTD “Multi-<br>Profile Clinic Consilium<br>Medulla”<br>6g, Politkovskaia Str., Tbilisi,<br>Georgia 0186	
Georgia	268008	Independent Ethics<br>Committee of LTD "Institute<br>of Clinical Oncology"<br>5, Lubliana str., Tbilisi,<br>Georgia 0159	
Greece	300003		Ministry of Health<br>284 Mesogion Ave<br>Cholargos, Greece 15562
Greece	300004		Ministry of Health<br>284 Mesogion Ave<br>Cholargos, Greece 15562
Greece	300007		Ministry of Health<br>284 Mesogion Ave

			Cholargos, Greece 15562
Greece	300008		Ministry of Health<br>284 Mesogion Ave<br>Cholargos, Greece 15562
Greece	300010		Ministry of Health<br>284 Mesogion Ave<br>Cholargos, Greece 15562
Hungary	348001		Egészségügyi Tudományos Tanács Klinikai Farmakológiai Etikai Bizottsága<br>Arany Janos u. 6-8<br>Budapest, Hungary 1051
Hungary	348002		Egészségügyi Tudományos Tanács Klinikai Farmakológiai Etikai Bizottsága<br>Arany Janos u. 6-8<br>Budapest, Hungary 1051
Jordan	400003	Institutional Review Board<br>Ibn Khaldoun Street Amman, Amman,<br>Jordan 11183	
Lebanon	422001	Institutional Review Board of Hammoud<br>Ground Floor, Dr. Ghassan Hammoud Street. Saida, South Lebanon, Lebanon	
Lebanon	422004	Institutional Review board of Ain Wazein<br>Medical Village<br>El chouf, Mount Lebanon, Lebanon	
Malaysia	458001	Medical Research Ethics Committee<br>Lembah Pantal<br>Kuala Lumpur, Kuala Lumpur, Malaysia,<br>59100	
Malaysia	458002		Medical Research & Ethics Committee<br>Blok A, Kompleks Institut Kesihatan, No 1, Jalan Setia Murni,U13/52, Seksyen U13, Bandar Setia Alam, 40170<br>Shah Alam Selangor, Malaysia 40170
Malaysia	458003		Medical Research & Ethics Committee<br>Blok A, Kompleks Institut Kesihatan, No 1, Jalan Setia Murni,U13/52, Seksyen U13, Bandar Setia Alam, 40170<br>Shah Alam Selangor, Malaysia 40170

Malaysia	458004	Sekretariat Ethika Penyelidikan University Kebangs<br>Tigkat 1, Blok Klinikal, Hospital Canselor Tuanku Muhriz ,Pusat Perubatan UKM Jalan Yaacob Latif, Bandar Tun Razak, Cheras, Kuala Lumpur, Malaysia 56000	
Malaysia	458005		Medical Research & Ethics Committee<br>Blok A, Kompleks Institut Kesihatan, No 1, Jalan Setia Murni,U13/52, Seksyen U13, Bandar Setia Alam, 40170 Shah Alam Selangor, Malaysia 40170
Malaysia	458006		Medical Research & Ethics Committee<br>Blok A, Kompleks Institut Kesihatan, No 1, Jalan Setia Murni,U13/52, Seksyen U13, Bandar Setia Alam, 40170 Shah Alam Selangor, Malaysia 40170
Mexico	484002	Comite de Investigacion del Hospital Universitario "Dr. Jose Eleuterio Gonzalez"<br>Francisco Ignacio Madero y avenida Gonzalitos, s/n, Monterrey, Nuevo León Mexico 64460	
Mexico	484004	Comite de Etica en Investigacion Mexico Centre for clinical Research SA de CV<br>Amores No. 709, Col del Valle. Delegacion Benito Juarez, Mexico City, Mexico 3100<br><br>Comite de Etica en Investigacion Clinica del sanatorio Alcocer Pozo SA de CV<br>Reforma No. 23<br>Santiago de Querétaro, Queretaro, Mexico<br>76000	
Mexico	484005	Comite de Etica en Investigacion Accelerium S de RL de CV<br>Calle Modesto Arreola No.917 Ote. Colonia Centro,	

		Monterrey, Nuevo León, Mexico<br>64000<br><br>Comite de Etica en Investigacion Clinica del sanatorio Alcocer Pozo SA de CV<br>Reforma No. 23<br>Santiago de Querétaro, Queretaro, Mexico<br>76000	
Mexico	484007	Comité de Investigación del Hospital Mision<br>Avenida del Hospital 112, 1º y 2º piso, Col. Sertoma, Sertoma, Monterrey, Mexico<br>64718	
Mexico	484008	Comité de Ética en Investigación de Investigación Biomédica para el Desarrollo de Fármacos S.A. de C.V.<br>Avenida Sebastián Bach No. 5257, Col. La Estancia, Zapopan, Jalisco, Mexico<br>45030	
Mexico	484009	Comité de Investigación del Hospital Mision<br>Avenida del Hospital 112, 1º y 2º piso, Col. Sertoma, Sertoma, Monterrey, Mexico<br>64718<br><br>Comite de Etica en Investigacion Clinica del sanatorio Alcocer Pozo SA de CV<br>Reforma No. 23<br>Santiago de Querétaro, Queretaro, Mexico<br>76000	
Mexico	484010	Comité de Ética en Investigación de Oaxaca Site Management Organization S.C.<br>Humboldt No. 302, Col. Centro Oaxaca, Oaxaca, Mexico 68000<br><br>Comité de Investigación<br>Humboldt No. 302, Col. Centro, Oaxaca, Mexico<br>68000	

Mexico	484011	Comité de Ética en Investigación del Centro Estatal de Cancerología Ejercito Mexicano 3700, Col. Centro Chihuahua, Chihuahua, Mexico 31000<br><br>Comité de Investigación Ejercito Mexicano 3700, Col. Centro<br>Chihuahua, Chihuahua, Mexico 31000	
Philippines	608003	Unified Research Ethics Review Committee<br>West Visayas State University, Luna St, La Paz, Iloilo City, Philippines 5000<br><br>Dr. Pablo O. Torre Memorial Hospital - Research Ethics Committee<br>B.S. Aquino Drive<br>Bacolod City, Negros Occidental, Philippines 6100	
Philippines	608005	IERC Davao Doctors Hospital 4th Floor Dr. Herminio A. Villano Sr. Oncology Center, 118 E. Quirino Avenue, Davao City, Philippines 8000	
Philippines	608008	Institutional Review Board - Manila Doctors Hospital<br>8/F Norberti Ty Medical Tower 2, 664 T.M. Kalaw Avenue, Ermita, Manila, Philippines 1000	
Philippines	608010	Lung Center of the Philippines nstitutional Ethics Review Board<br>4th Floor Room 4013 Lung Center of the Philippines, Quezon Avenue Ext. Barangay Maharlika, Quezon City, Philippines 1100	
Poland	616002		Komisja Bioetyczna<br>ul. Sniadeckich 33<br>Gdansk, Poland 80 204
Poland	616003		Komisja Bioetyczna<br>ul. Sniadeckich 33<br>Gdansk, Poland 80 204
Poland	616004		Komisja Bioetyczna<br>ul. Sniadeckich 33<br>Gdansk, Poland 80 204
Poland	616005		Komisja Bioetyczna<br>ul. Sniadeckich 33

			Gdansk, Poland 80 204
Poland	616006		Komisja Bioetyczna<br>ul. Sniadeckich 33<br>Gdansk, Poland 80 204
Poland	616007		Komisja Bioetyczna<br>ul. Sniadeckich 33<br>Gdansk, Poland 80 204
Poland	616008		Komisja Bioetyczna<br>ul. Sniadeckich 33<br>Gdansk, Poland 80 204
Poland	616011		Komisja Bioetyczna<br>ul. Sniadeckich 33<br>Gdansk, Poland 80 204
Poland	616012		Komisja Bioetyczna<br>ul. Sniadeckich 33<br>Gdansk, Poland 80 204
Romania	642003		National Bioethics Committee of Medicines and Medical Devices<br>Sos. Stefan cel Mare no. 19-21 ,District 2<br>Bucharest, Romania 020125
Romania	642004		National Bioethics Committee of Medicines and Medical Devices<br>Sos. Stefan cel Mare no. 19-21 ,District 2<br>Bucharest, Romania 020125
Romania	642005		National Bioethics Committee of Medicines and Medical Devices<br>Sos. Stefan cel Mare no. 19-21 ,District 2<br>Bucharest, Romania 020125
Russia	643001	Ethics Committee of State Budgetary Institution Sverdlovsk Regional Oncology Dispensary<br>29, Soboleva Str.<br>Yekaterinburg, Sverdlovsk Region, Russia 620036	
Russia	643002	Ethics Committee of Arkhangelsk Clinical Oncological Dispensary<br>Bld.1, 145, Obvodniy Kanal Prospekt<br>Arkhangelsk, Arkhangelsk Region, Russia 163045	
Russia	643003	Local Ethics Committee of EVIMED LLC<br>22, 9-v, Blyukhera Str.<br>Chelyabinsk, Chelyabinsk Region, Russia 454048	
Russia	643005	Ethics Committee of the federal state-funded	

		institution “National Medical Science Centre of Oncology n. a. N.N. Blokhin” of the Ministry of Health of the Russian Federation<br>24, Kashirskoe Shosse<br>Moscow, Russia 115478	
Russia	643007	Ethics Committee of State Budgetary Healthcare Institution Regional Clinical Oncology Dispensary<br>35, Volgogradskaya Str., Kemerovo, Kemerovo Region, Russia 650036	
Russia	643008	Ethics Committee at Private Institution Reaviz Medical University<br>100, Chkalova Str<br>Samara, Samara Region, Russia 443030	
Russia	643010	Committee for the Ethical Review of Clinical Studies at Regional State Budgetary Healthcare Institution Belgorod Regional Oncology Center, 1, Kuybysheva Str., Belgorod, Russia 308010	
Russia	643011	Local Ethics Committee at the Regional Budget Healthcare Institution “Kursk Regional Clinical Oncology Dispensary”<br>20, Pirogova Str., Kursk, Russia 305035	
Russia	643013	Local Ethics Committee of FSBfI N.N. Petrov MRC of Oncology of Russian MoH<br>68, Leningradskaya Str., Pos. Pesochny<br>Saint Petersburg, North-West Federal District, Russia 197758	
Russia	643014	EC at the State Budgetary Healthcare Institution of the Kaluga Region “Kaluga Regional Clinical Oncology Dispensary”<br>2, Vishnevskogo Str.<br>Kaluga, Russia 248007	
Russia	643015	Local Ethics Committee of State Budgetary Dispensary of the Ministry of Healthcare of the Republic of Bashkortostan<br>73/1, Prospekt Oktyabrya	

		Ufa, Republic of Bashkortostan, Russia 450054	
Russia	643016	Local Ethics Committee at The Federal State Budgetarty Institution of Higher Education “N.P. Ogarev Mordovia State University” 26A, Ulyanova Str Saransk, Republic of Mordovia, Russia 430032	
Russia	643017	Ethics Committee at State Funded Healthcare Institution of Moscow “Moscow City Oncology Hospital No. 62” of the Moscow Department of Health Bld. 1-26, 27, pos. Istra, Krasnogorskiy District, Moscow, Russia 143423<br><br>Independent Interdisciplinary Committee for the Ethical Review of Clinical Trials Leningradsky pr-t, 51 Stepanovskoe PO, Moscow Region, Russia 143423	
Russia	643018	Independent Ethics Committee of at Arte Med Assistance Ltd. office 44, buil. C, 27, Engelsa ave. Saint Petersburg, Russia 131156	
Russia	643019	Ethics Committee of 'Komanda' LLC Room 370N, Lit. A, ,Bld. 2, 19 Frunze Str. Saint Petersburg North-West Federal District, Russia 196135	
Russia	643020	Ethics Committee of Saint-Petersburg State 'City Clinical Oncology Dispensary' 56, pr. Veteranov Saint-Petersburg, North-West Federal District, Russia 198255	
Russia	643022	Ethics Committee of Budgetary Healthcare Institution of Omsk Region «Clinical Oncology Dispensary» Bld. 1, 9, Zavertyayeva str., Omsk, Russia 644013	

Russia	643023	Ethics Committee of Leningrad Region Clinical Oncology Dispensary<br>37-39 Liteyniy ave<br>Saint-Petersburg, North-West Federal District, Russia 191014	
Russia	643024	Ethics Committee of the State Autonomous Healthcare Institution “Republican Clinical Oncology Centre” of the Ministry of Healthcare of the Republic of Tatarstan<br>29, Sibirsky trakt<br>Kazan, Republic of Tatarstan, Russia 420029	
Russia	643027	Interuniversity Ethics Committee<br>Bld 1, 20, Delegatskaya str., 127473, Moscow, Russia	
Russia	643029	Ethics Committee of Pyatigorsk Interdistrict Oncology Centre<br>31, Kalinina str.<br>Pyatigorsk, Stavropol Region, Russia 357519	
Spain	724009		CEIm Hospital Universitario Gregorio Maranon<br>C/ Dr. Eszuerdo, 46, Madrid, Spain 28007
Thailand	764001	Ethic Committee of Udonthani Cancer Hospital<br>36 Moo 1, Udon-Khon Kaen Road ,Udonthani, Udonthani, Thailand 41330	
Thailand	764004	The Institutional Review Board, Royal Thai Army Medical Department<br>317/5 Rajavithi Road, Rajathevee, Bangkok, Thailand 10400	
Thailand	764007	Institutional Review Board, 1873 RamaIV Road, Patumwan, Bangkok, Thailand 10330	
Thailand	764008	Human Research Committee Prince of Songkla University ,15 Karnchanavanich Road Songkla, Thailand 90110	
Thailand	764009	The Internal Ethical Committee for Chiangrai Prachanukroh Hospital<br>1039 Sathanpayaban, Muang, Chiang Rai, Thailand 57000	

Thailand	764011	Ethics Committees on Researches Involving Human Subjects Rajavithi Hospital<br>2 Phyathai Road, Ratchathewi, Bangkok, Thailand 10400	Central Research Ethics Committee<br>The National Research Council of Thailand, Paholyothin Rd, Bangkhen, Bangkok, Thailand
Thailand	764012	The Ethics Committee, Lampang Cancer Hospital<br>199 Lampang-Chiangrai Rd.Rd., Pichai, Muang, Lampang, Thailand 52000	
Turkey	792002		Ankara Universitesi Tip Fakultesi Dekanligi, Klinik Arastirmalar Ktik Kurulu, Sihhiye, Ankara, Turkey 06100
Turkey	792003		Ankara Universitesi Tip Fakultesi Dekanligi, Klinik Arastirmalar Ktik Kurulu, Sihhiye, Ankara, Turkey 06100
Turkey	792004		Ankara Universitesi Tip Fakultesi Dekanligi, Klinik Arastirmalar Ktik Kurulu, Sihhiye, Ankara, Turkey 06100
Turkey	792005		Ankara Universitesi Tip Fakultesi Dekanligi, Klinik Arastirmalar Ktik Kurulu, Sihhiye, Ankara, Turkey 06100
Turkey	792006		Ankara Universitesi Tip Fakultesi Dekanligi, Klinik Arastirmalar Ktik Kurulu, Sihhiye, Ankara, Turkey 06100
Turkey	792007		Ankara Universitesi Tip Fakultesi Dekanligi, Klinik Arastirmalar Ktik Kurulu, Sihhiye, Ankara, Turkey 06100
Turkey	792008		Ankara Universitesi Tip Fakultesi Dekanligi, Klinik Arastirmalar Ktik Kurulu, Sihhiye, Ankara, Turkey 06100
Turkey	792009		Ankara Universitesi Tip Fakultesi Dekanligi, Klinik Arastirmalar Ktik Kurulu, Sihhiye, Ankara, Turkey 06100
Turkey	792010		Ankara Universitesi Tip Fakultesi Dekanligi, Klinik Arastirmalar Ktik Kurulu, Sihhiye, Ankara, Turkey 06100
Turkey	792011		Ankara Universitesi Tip Fakultesi Dekanligi, Klinik Arastirmalar Ktik Kurulu, Sihhiye, Ankara, Turkey 06100
Turkey	792012		Ankara Universitesi Tip Fakultesi Dekanligi, Klinik Arastirmalar Ktik Kurulu, Sihhiye, Ankara, Turkey 06100

Turkey	792013		Ankara Universitesi Tip Fakultesi Dekanligi, Klinik Arastirmalar Ktik Kurulu, Sihhiye, Ankara, Turkey 06100
Turkey	792014		Ankara Universitesi Tip Fakultesi Dekanligi, Klinik Arastirmalar Ktik Kurulu, Sihhiye, Ankara, Turkey 06100
Turkey	792015		Ankara Universitesi Tip Fakultesi Dekanligi, Klinik Arastirmalar Ktik Kurulu, Sihhiye, Ankara, Turkey 06100
Turkey	792016		Ankara Universitesi Tip Fakultesi Dekanligi, Klinik Arastirmalar Ktik Kurulu, Sihhiye, Ankara, Turkey 06100
Ukraine	804001	Ethics Committee of MI "City Dnipropetrovsk Multi-field Clinical Hospital #4"<br>31, Blizhniaya Str.31, Dnipro, Ukraine 49102	
Ukraine	804002	Ethics Committee of MI of KRC "Kyiv Regional Oncological Dispensary"<br>1A, Baggovutivska Str.<br>Kyiv, Ukraine 04107	
Ukraine	804003	Ethics committee of Communal Non-Profit Enterprise "Zaporizhzhya Regional Antitumor Center" of Zaporizhzhya Regional Council<br>177A, Kulturna Str.<br>Zaporizhzhya, Ukraine 69040	
Ukraine	804004	Ethics committee of National Institute of Cancer<br>33/43, Lomonosova str., Kyiv, Ukraine 03022	
Ukraine	804006	Ethics Committee of Municipal non-profit enterprise "Regional Oncology Center"<br>4, Lisoparkivska Str.<br>Kharkiv, Ukraine 61070	
Ukraine	804008	Ethics committee of a private enterprise private production firm "ACINUS"<br>65, Velyka Perspektyvna Str.<br>Kropyvnytskyi, Ukraine 25006	
Ukraine	804009	Ethics Committee of Communal Noncommercial Enterprise "Kherson Regional	

		Oncological Dispensary" of Kherson regional council<br>26b Vyacheslava Chornovola Highway, Antonivka village, Kherson, Ukraine 73035	
Ukraine	804010	Ethics committee of medical center «medical center of yuriy spizhenko» LLC<br>21B, vul. Soborna Str., Kapitanivka, Kyiv-Sviatoshyno<br>Kyiv, Ukraine 08112	
Ukraine	804011	Ethics Committee of Zakarpatiia Regional Clinical Oncological Dispensary<br>2, Brodlakovycha Str.<br>Uzhgorod, Ukraine<br>88014	
Ukraine	804012	Ethics Committee of Vinnitsa Regional Clinical Oncological Center<br>84, Khmelnytske Highway, Vinnytsia, Ukraine 21029	
Ukraine	804013	Ethics Committee of Medical and Diagnostic Center of LLC "Specialized Clinic "GOOD PROGNOSIS"<br>40-A, Vatslava Gavela Boulevard, Kyiv, Ukraine 03126	
